# Ecogenomic survey of plant viruses infecting Tobacco by Next generation sequencing

**DOI:** 10.1186/s12985-016-0639-7

**Published:** 2016-11-04

**Authors:** Ibukun A. Akinyemi, Fang Wang, Benguo Zhou, Shuishui Qi, Qingfa Wu

**Affiliations:** 1 School of Life Sciences, University of Science and Technology of China, Hefei, Anhui 230027 China; 2 Tobacco Research Institute, Anhui Academy of Agricultural Sciences, Hefei, Anhui 230031 China

**Keywords:** sRNA, Bioinformatics, Assembly, Next generation sequencing, Plant virus

## Abstract

**Background:**

The invasion of plant by viruses cause major damage to plants and reduces crop yield and integrity. Devastating plant virus infection has been experienced at different times all over the world, which are attributed to different events of mutation, re-assortment and recombination occurring in the viruses. Strategies for proper virus management has been mostly limited to eradicating the vectors that spreads the plant viruses. However, development of prompt and effective diagnostic methods are required to monitor emerging and re-emerging diseases that may be symptomatic or asymptomatic in the plant as well as the genetic variation and evolution in the plant viruses. A survey of plant viruses infecting field-grown Tobacco crop was conducted in Anhui Province of China by the deep sequencing of sRNAs.

**Methods:**

Survey of plant viruses infecting Tobacco was carried based on 104 samples collected across the province. Nine different sRNA libraries was prepared and custom-made bioinformatics pipeline coupled with molecular techniques was developed to sequence, assemble and analyze the siRNAs for plant virus discovery. We also carried out phylogenetic and recombination analysis of the identified viruses.

**Results:**

Twenty two isolates from eight different virus species including *Cucumber mosaic virus*, *Potato virus Y*, *Tobacco mosaic virus*, *Tobacco vein banding Mosaic virus*, *Pepper mottle virus*, *Brassica yellow virus*, *Chilli venial mottle virus*, *Broad bean wilt virus 2* were identified in tobacco across the survey area. The near-complete genome sequence of the 22 new isolates were determined and analyzed. The isolates were grouped together with known strains in the phylogenetic tree. Molecular variation in the isolates indicated the conserved coding regions have majorly a nucleotide sequence identity of 80-94 % with previously identified isolates. Various events of recombination were discovered among some of the isolates indicating that two or more viruses or different isolates of one virus infect the same host cell.

**Conclusion:**

This study describes the discovery of a consortium of plant viruses infecting Tobacco that are broadly distributed in Anhui province of China. It also demonstrates the effectiveness of NGS in identifying plant viruses without a prior knowledge of the virus and the genetic diversity that enhanced mixed infection.

**Electronic supplementary material:**

The online version of this article (doi:10.1186/s12985-016-0639-7) contains supplementary material, which is available to authorized users.

## Background

Viruses are ubiquitous and affects plant growth and yield. Over the years, crop losses due to viral infection have been very devastating and are of great concern especially in developed and developing countries [1]. The unrestricted distribution of plant diseases and emerging infectious disease poses serious threat to food sustenance. A high percentage of this loss is caused by viruses because of their abundant presence in most environments among the biological entities [2]. Early identification of these plant pathogens remains the focal point in the field of virology, aimed at preventing the spread of the viruses as well as developing ways of combating and reducing their effects on agricultural yield.

RNA viruses exists within host as a consortium of un-identical but similar sequences due to their inability to propagate without a living host, high mutation and recombination rates, referred to as viral quasispecies [3]. RNA silencing is one of the defense mechanisms of plant against viruses, in which the double stranded RNA (dsRNA) serves as a substrates for Dicer-like ribonuclease (DCL) to produce small-interfering RNAs (siRNAs) of between 21 to 25 nt [4]. Viral infection of plants involves the production of viral small RNAs (vsRNAs) and the plant host interacts with invading viruses by developing various cellular mechanisms. Viruses are both inducers and targets of RNAi [5]. Double stranded RNA intermediates are produced by viral genomes during replication which serves as substrates for Dicer-like ribonucleases and cleaves into small virus derived siRNAs (viRNAs), and binds with the Argonaut protein (Ago) to form the RISC complex [6].

The advent of new sophisticated technologies for parallel sequencing had increased our understanding of viral genome variability and evolution within the host and virus defense mechanism in plants. It is widely accepted that studies of viral abundance and diversity will lead and have led to novel insights into the functioning of the microbial biosphere. The relative abundance of a virus (or viral nucleic acid) in a sample, compared to that of other organisms such as bacteria or host cells (or their genomes), is a critical factor for the discovery of viruses when using metagenomics. Unlike traditional virus detection methods e.g., enzyme-linked immunosorbent assay (ELISA), polymerase chain reaction (PCR), or microarray which depends on prior knowledge of antibody or sequence of the potential virus [7] as well as determining the existence of novel viral agents [8, 9], the use of next generation sequencing technology (NGS) provides a powerful method for determining the causative pathogen without the prior knowledge of the disease pathogen. The genome of plant viruses can be rapidly determined even when occurring at extremely low titers in the infected host. The detection of both DNA and RNA viruses [10] has been made possible by the reconstruction of partial or complete viral genomes [11] and sequencing of the accumulated 21–24 nt virus-derived siRNAs generated by Dicer enzymes upon recognition of viral dsRNA. With the development of NGS technology and its relatively low cost, NGS has widen understanding and its potentials of diagnostics of viral pathogens without a priori knowledge of the invading pathogen, which provide accurate and timely detection of these viral pathogens in plant for effective disease management and control. Consequently, it is necessary to conduct an accurate and timely detection of these viral pathogens in plant for effective disease management and control.

Tobacco (*Nicotiana tabacum*) is an important economic crop worldwide, with half of the world’s tobacco farmers in China and the world’s largest producer [12], ahead of countries such as India, Zimbabwe, Indonesia, Turkey, Bangladesh, Egypt, Philippine and Thailand. The production and yield of tobacco have been seriously affected by the invasion of emerging and recurrent plant viruses with symptoms such as venial necrosis, mosaic, mottling, yellowing, ring spots, stunting, shoestring and deformation [13–16]. Anhui province is in the center of China and surrounded by six other provinces. The typical geographical feature of this area is enriched with geochemical elements suitable for the flourishing of Tobacco plant [17]. The tobacco plantation is usually surrounded or mixed with other crops, as mixed system of farming is commonly adopted in these areas. These however, had enhanced the transmission of plant virus from one plant to the other and consequently made Anhui province an idealistic open ecosystem to investigate the viruses infecting the crop in the province. In order to identify the etiological agents of the different disease symptoms observed in different Tobacco plantation across Anhui province, we used next generation sequencing of small RNAs to identify viruses from symptomatic Tobacco plants in farm fields. We also present the results of genome comparison between the resulting 22 isolates and genomes retrieved from GenBank. This study provides census of viral population and distribution in different ecosystem or cropping system through the characterization, discovery and molecular interaction of plant viruses. We also described the recombination events that occurred in the isolates and a bioinformatics pipeline that explores the siRNA generated in response to viral invasion and other molecular biology methods employed to discover a consortium of viruses infecting tobacco.

## Results

### Sequence data

One hundred and four symptomatic leaf samples of tobacco were collected from farm fields and pooled into 9 combinations based on the location of sample collected. Nine individual sRNA libraries were constructed and sequenced on an Illumina HiSeq-2000 platform which generated between 38 and 49 million raw reads. Adapters and low quality reads were trimmed to obtain between 32 and 45 million clean reads with lengths of 18–30 nt (Tables 1). The most abundant class of viral derived small RNAs were 21–24 nt in length, with a peak of 24 nt for all samples (Additional file 1: Figure S1). Similar length distribution of sRNA were observed in all the libraries (Additional file 1: Figure S2a,b,c).

**Table 1:** Description of Next generation sequencing data from one hundred and four samples

Library Source (City)	Library Name	No of Samples	Raw Reads	Clean reads	Unique Seq	No of Contigs Assembled by Velvet (*k = 17*)
Xuancheng	Xch 1	14	44,729,605	38,973,127	7,051,733	7952
	Xch 2	11	49,938,373	45,290,836	7,131,823	7038
Xuancheng/Wuhu	Xch-Wh	5	38,747,172	34,282,436	5,985,886	6346
Dongzhi/Qingyang	Dzh-Qyg	11	43,827,189	38,882,338	6,522,149	6776
Bozhou	Bzh 1	17	45,974,903	39,218,493	7,582,592	8133
	Bzh 2	11	41,574,669	36,303,201	7,641,767	8981
	Bzh 3	9	43,324,495	37,668,456	6,579,416	7388
	Bzh 4	8	41,720,630	37,414,819	6,770,175	8011
	Bzh 5	18	38,277,531	32,358,941	6,088,147	6698

### Virus identification

**Fig. 1:**
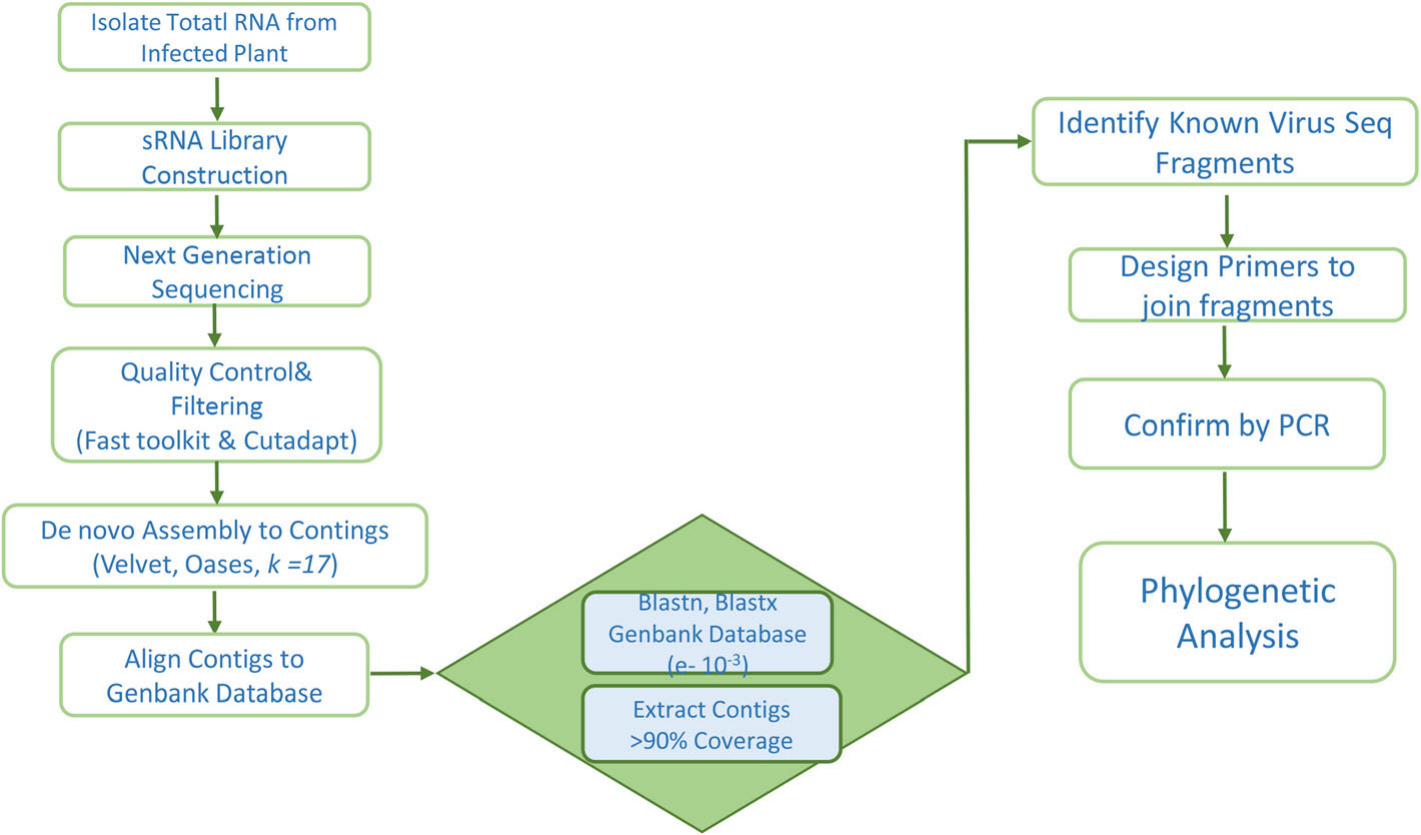
Schematic representation of virus detection and discovery pipeline using next generation sequencing

The homology-dependent approach for plant virus discovery was employed for the analysis, using a custom-made pipeline (Fig. 1). The clean reads of each library were assembled into contigs with Velvet [18] and Oases [19]. The number of contigs produced were 6346–8981 (Table 1) based on the *de-bruijn* graph algorithm (*k -mer* 17), with the longest single contig length of 2,255 nt. GC content of the assembled contigs ranges between 43 and 45 %. These contigs were searched against the non-redundant nucleotide sequence entries of the National Center for Biotechnology Information (NCBI) database by BLASTn with e-value of 10^−3^. Stringent parameters of ≥ 90 % identity and coverage identified 554 contigs hit distantly related to sequences in the database at nucleotide and protein level (Table 2). Related reference genome sequences were used to determine the relative positions and orientation of these final contigs. Subsequently, RT-PCR was performed to obtain the near-complete genome sequence of all the isolates using primers designed based on the final contigs. The genome sequence of identified viruses from all the libraries have high nucleotide sequence identity with respective matching genome from the Genbank database (Table 3). BLASTn search of the full-length nucleotide sequence against the database indicated the assembled genomes from the libraries have significant similarities to viruses belonging to Cucumovirus, Tobamovirus, Polerovirus and Potyvirus of the Family Bromoviridae, Virgaviridae, Luteoviridae and Potyviridae respectively (Table 3). Specifically, 8 different virus species were identified which includes *Cucumber mosaic virus* (CMV), *Potato virus Y* (PVY), *Tobacco mosaic virus* (TMV), *Tobacco vein banding Mosaic virus* (TVBMV), *Pepper mottle virus* (PeMV), *Brassica yellow virus*, *Chilli venial mottle virus* (CVMV), *Broad bean wilt virus 2* (BBWV) infecting tobacco across the survey area. In order to gain insight into the host RNA silencing mechanism induced by the invading viruses, analysis of the sequences characterized by Bowtie [20] allowing no mismatch showed an equal distribution of siRNAs along the genome of the new virus while no excess of (+) over (−) siRNAs (Additional file 1: Figure S3) but revealed a peak of 22 and 24 nts in the genome sequence. The genome sequence of the 22 isolates from this study has been deposited at the GenBank database under the accession number KX650847-KX650868.

**Table 2:** Analysis of the assembled contigs against sequences in the NCBI database, using a highly homology sequence search

Library Source	Library Name	Viruses	No of contigs identical to Blastn	Size (nt)	G + C %	Mapped Organism	Relative (ref Seq)
Xuancheng	Xch 1	CMV-Xch-1	43	2874	45.95	Cucumber Mosaic Virus	3357
		PVY-Xch-1	20	9292	42.53	Potato Virus Y	9704
		PeMV-Xch	14	9632	43.42	Pepper Mottle Virus	9640
	Xch 2	CMV-Xch-2	24	2922	44.92	Cucumber Mosaic Virus	3357
		TMV-Xch-2	23	4679	42.87	Tobacco Mosaic Virus	6395
Xuancheng/Wuhu	Xch-Wh	CMV-Xch-Wh	5	3102	45.34	Cucumber Mosaic Virus	3357
		PVY-Xch-Wh	14	9302	42.02	Potato Virus Y	9704
		BrYV-Xch-Wh	12	3494	42.86	Brassica Yellow Virus	5666
		TMV-Xch-Wh	31	5808	43.10	Tobacco Mosaic Virus	6395
Dongzhi/Qingyang	Dzh-Qyg	TMV-Dzh-Qyg	33	5571	43.10	Tobacco Mosaic Virus	6395
		CVMV-Dzh-Qyg	20	9499	41.08	Chilli venial mottle virus	9711
Bozhou	Bzh-1	PVY-Bzh-1	21	8826	42.18	Potato Virus Y	9704
	Bzh-2	CMV-Bzh-2	29	2786	38.42	Cucumber Mosaic Virus	3357
	Bzh-3	PVY-Bzh-3	34	9560	42.46	Potato Virus Y	9704
		CMV-Bzh-3	18	2754	45.51	Cucumber Mosaic Virus	3357
		TvBMV-Bzh-3	41	9148	42.15	Tobacco Vein Banding Mosaic virus	9570
	Bzh-4	PVY-Bzh-4	37	9412	42.11	Potato Virus Y	9704
		CMV-Bzh-4	16	2836	45.89	Cucumber Mosaic Virus	3357
		TvBMV-Bzh-4	31	9449	42..12	Tobacco Vein Banding Mosaic virus	9570
	Bzh-5	CMV-Bzh-5	24	2967	46.22	Cucumber Mosaic Virus	3357
		BBWV2-Bzh-5	29	3106	43.59	Broad Bean Wilt Virus 2	3607
		TvBMV-Bzh-5	35	9312	41.97	Tobacco Vein Banding Mosaic virus	9570
		Total=	554				

Note: Reference used were retrieved from the NCBI database *Cucumber mosaic virus* (NC_002034.1), *Potato virus Y(NC_001616.1)*, *Tobacco mosaic virus(NC_001367.1)*, *Tobacco vein banding Mosaic virus(NC_009994.1)*, *Pepper mottle virus(NC_001517.1)*, *Brassica yellow virus(NC_016038.1)*, *Chilli venial mottle virus(NC_005778.1)*, *Broad bean wilt virus 2*(NC_003004.1)

**Table 3:** Percentage nucleotide identity of identified viruses in each library to reference genome from NCBI database

Virus Identified	Xch 1	Xch 2	Xch-Wh	Dzh-Qyg	Bzh 1	Bzh 2	Bzh 3	Bzh 4	Bzh 5
Cucumber mosaic virus	77.34	77.58	84.63	–	–	62.64	75.07	77.95	80.25
Potato virus Y	89.15	–	84.78	–	81.67	–	83.88	88.00	–
Tobacco Mosaic virus	–	72.06	89.31	86.11	–	–	–	–	–
Tobacco Vein Banding Mosaic virus	–	–	–	–	–	–	87.06	89.40	88.61
Pepper Mottle Virus	94.18	–	–	–	–	–	–	–	–
Chilli venial mottle virus	–	–	–	78.70	–	–	–	–	–
Brassica Yellow Virus	–	–	52.97	–	–	–	–	–	–
Broad Bean Wilt Virus 2	–	–	–	–	–	–	–	–	72.48

–: Not available

### Genome properties

The single protein gene 1a responsible for viral replication was predicted in the CMV isolates Xch-1, Xch-2, Xch-Wh, Bzh-2, Bzh-3, Bzh-4, Bzh-5 (accession number KX650847, KX650848, KX650849, KX650850, KX650851, KX650852, KX650853 respectively) by the ORF Finder. The gene is a major virulence factor of the cucumber mosaic virus (CMV) and its essential for replication of the viral genome [21]. The ORF 1a encodes 993 amino acids (aa) protein, known to be involved in replication. The conserved domain of the putative ORF 1a-encoded protein of the CMV isolates are also found in association with two enzymatic motifs: viral methyl transferase and viral helicase consistent with other cucumoviruses [22]. Also, a large ORF 1a of the CMV isolates encoded the virus replicase protein of between 533 and 933 amino acids (aa) with an estimated molecular mass of ~ 45–112 kDa consistent with other cucumoviruses. The 7 isolates of the CMV have high nucleotide similarity with the reference genome of isolate Fny (D00356). CMV-Xch-1 (KX650847) shares 79 % nucleotide sequence identity with CMV-Ix isolate from the Philippines (U202201), while CMV-Bzh-4 (KX650852) had 80 % identity with the CMV-SD isolate (AF071551) from China.

The PVY isolates contain a single large ORF predicted by ORF Finder, consistent with other members of the potyvirus. The ORF of PVY-Xch-1, PVY-Xch-Wh, PVY-Bzh-1 isolates (accession number KX650858, KX650859, KX650860) are 166–7,362, 2,568–9,095 and 2,723–8,824 nt and encodes a polyprotein of 2,398, 2,175 and 2,033 aa with an estimated molecular mass of ~ 272, 248 and 231 kDa respectively. Comparison of PVY isolates polyprotein aa sequence with those of other PVY strains showed the presence of nine predicted cleavage sites, generating ten mature proteins (P1, HC-Pro, P3, 6 K1, CI, 6 K2, Vpg, NIa-Pro, NIb, and CP) after processing by virus-encoded proteases [23]. PVY-Bzh-1 (KX650860) has 90 % nucleotide identity with isolate PVY-Guiding-3 (HM590405), representing an a close similarity between the sequences. The ORF predicted in pepper mottle virus (PeMV-Xch) isolate (KX650857) reveals a single long polyprotein between 2,423 and 7,345 nt, also typical of potyviruses of the family Potyviridae [24]. PeMv-Xch encodes 1,640 aa, estimated to have a molecular weight of 186 kDa. This share 94 % nucleotide identity with the isolate (NC_001517.1) from USA.

Three isolates were identified to be Tobacco mosaic virus from samples collected in Xuancheng, Dongzhi, Qingyang and Bozhou. TMV has two replication-related genomic protein segment of 126 and 183 kDa, 30-kDa movement protein (MP) and a 17.5-kDa coat protein (CP). TMV-Xch-2 (KX650854) isolate encodes replicase protein between the 3 and 4,127 nt, and a movement protein encoded between 4,111 and 4,677 nt. Also the replicase and movement protein of TMV-Dzh-Qyg (KX650856) isolate are encoded between 144 and 4,585 nt and 4,569–5,375 nt respectively. TMV-Xch-Wh (KX650855) however has additional coat protein encoded between 5,410–5,808 nt expressed from a second subgenomic RNA [25].

### Phylogenetic analysis of Isolates

**Fig. 2:**
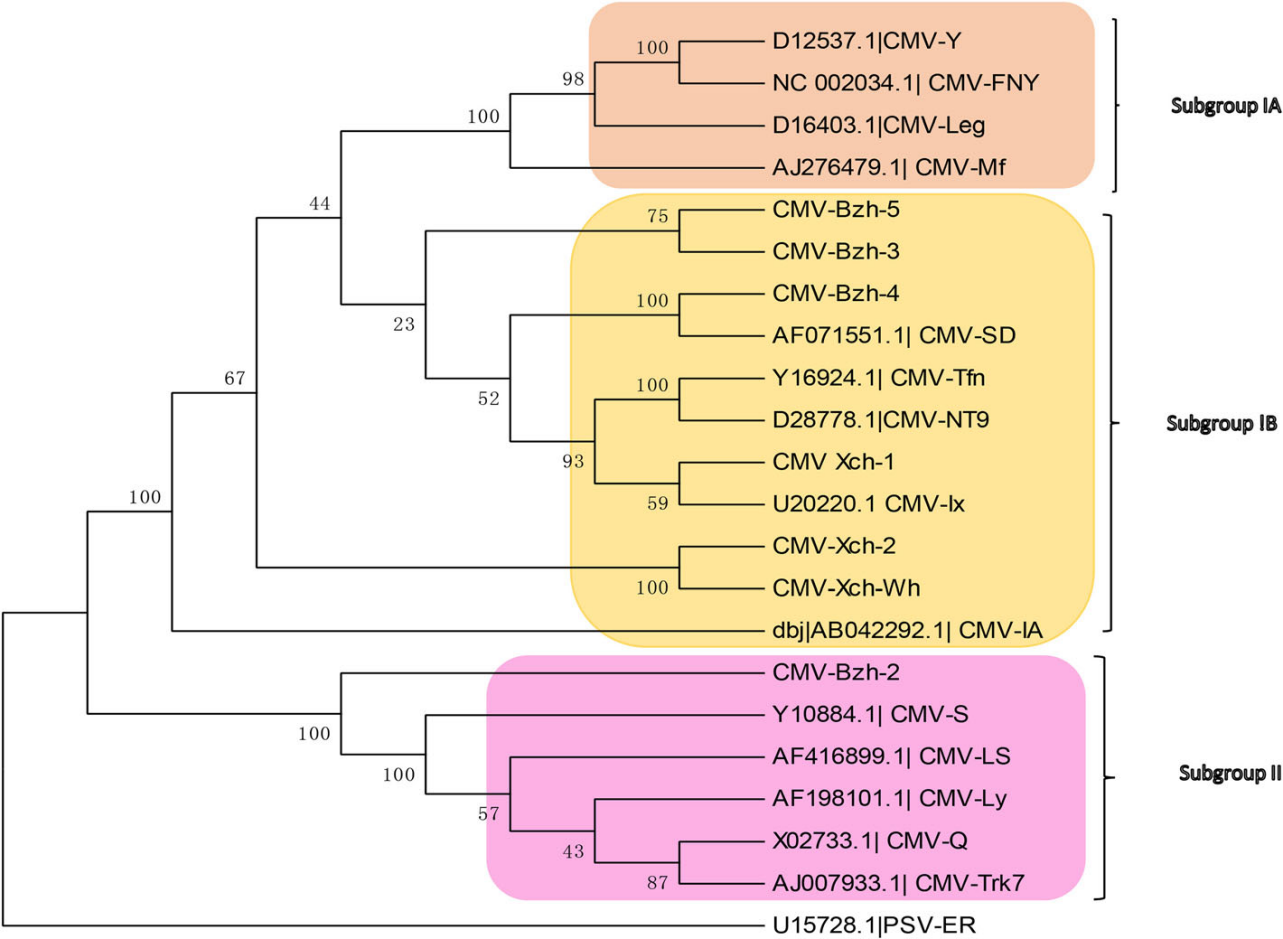
Phylogenetic analyses of Cucumber mosaic virus Isolates and selected strains, with aligned nucleotide sequences, generated using the neighbor-joining method and MEGA6 software. The percentage of replicate trees in which the associated taxa clustered together in the bootstrap test (1000 replicates) is shown next to the branches. Peanut stunt virus ER RNA 1 was used as an out group for the tree construction

The identified near-complete nucleotide sequences in this study, other previously identified isolates as well as major strains of species with large group members were retrieved from the GenBank database and used to determine the phylogenetic relationships between the isolates and members of the same genus. This is important to understand plant virus evolution and events such as recombination and reassortment that are major evolutionary force in virus population [26]. Neighbor-joining phylogenetic analysis based on full-length viral genomes was performed with a bootstrap of 1000 replications. The genomic sequence of the 7 CMV isolates (Table 2) shared a single 1a gene of between 80 and 95 % identity with other isolates. The isolates were grouped into clusters according to the sequences of the closest relatives in the genus (Fig. 2). All the selected sequences of the cucumoviruses and identified isolates were well separated into three subgroups (IA, IB and II). The new cucumber mosaic virus isolates from Anhui province, China formed clusters with some proven standard isolates that were taken as reference isolates for each subgroup such as isolates Fny, MF and Y for subgroup IA; isolates CTL, NT9, SD and TFN for subgroup IB; isolates LY, Q, S and Trk7 for subgroup II, which are consistent with previous studies [22, 27]. The divergence of subgroups I and II is clearly seen in the 1a phylogeny estimation (Fig. 2); however, further divergence of subgroup I into IA and IB is not obvious but indicates an evolutionary history that is quite different from what is seen in the other RNAs. The 87.24 % sequence identity of isolate CMV-Xch-1 (KX650847) and CMV-Xch-2 (KX650848) formed the same cluster in the subgroup 1b while the CMV-Bzh-3 (KX650851) and CMV-Bzh-5 (KX650853) isolates from different county in Bozhou form separate cluster with 90 % nucleotide identity in the same subgroup. However, the CMV-Bzh-4 (KX650852) had 90.12 % identity with the CMV-SD (AF071551.1) isolate while CMV-Bzh-2 is the only isolate in the subgroup II, indicating that most isolates from the China survey are clustered in subgroup 1 (Fig. 2).

**Fig. 3:**
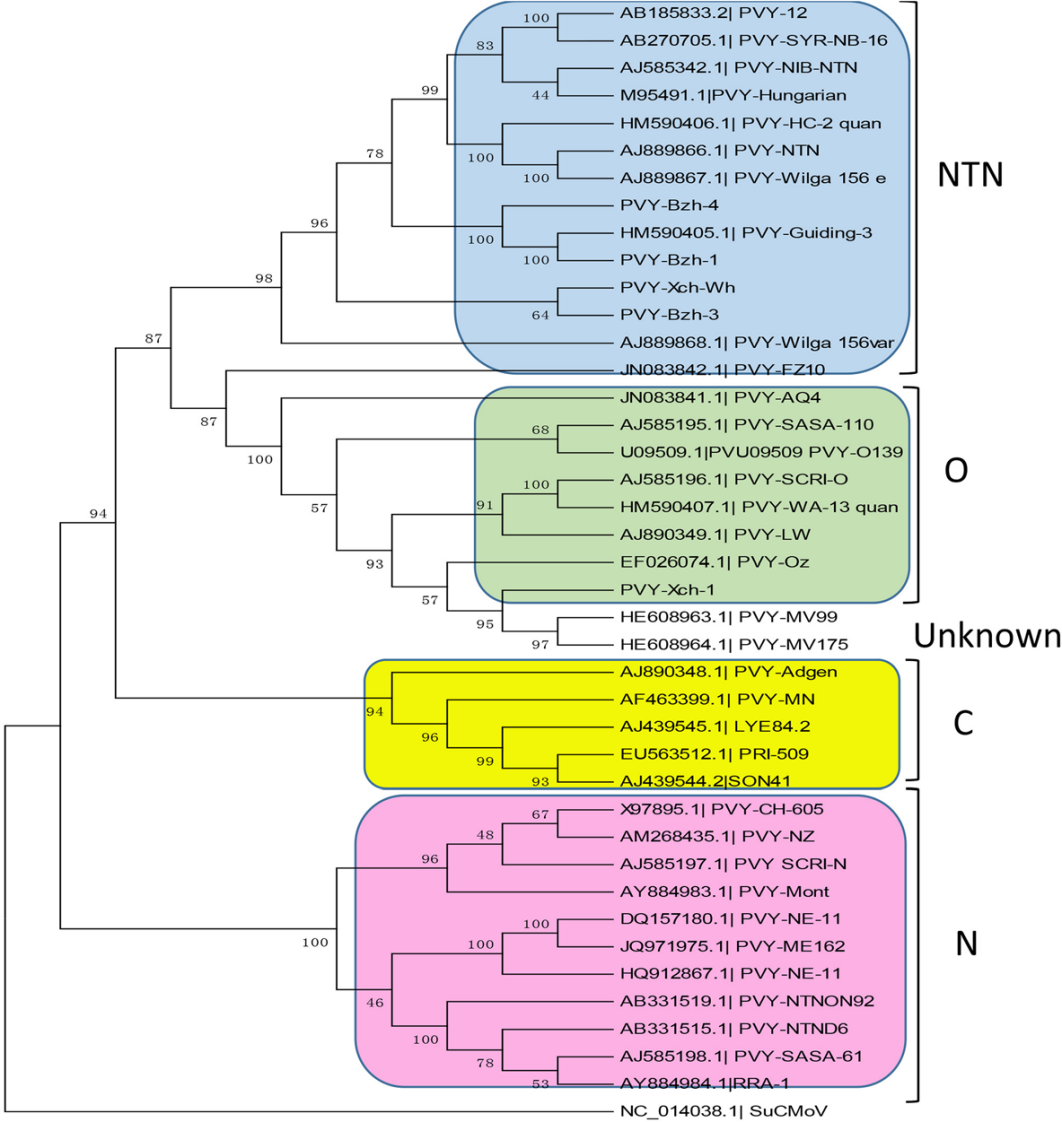
Phylogenetic analyses of Potato virus Y isolates and selected strains, with aligned nucleotide sequences, generated using the neighbor-joining method and MEGA6 software. The percentage of replicate trees in which the associated taxa clustered together in the bootstrap test (1000 replicates) is shown next to the branches. The tree of the Potyvirus was rooted using *Sunflower chlorotic mottle virus* (SuCMoV) as an out group sequence for the phylogenetic analysis

**Fig. 4:**
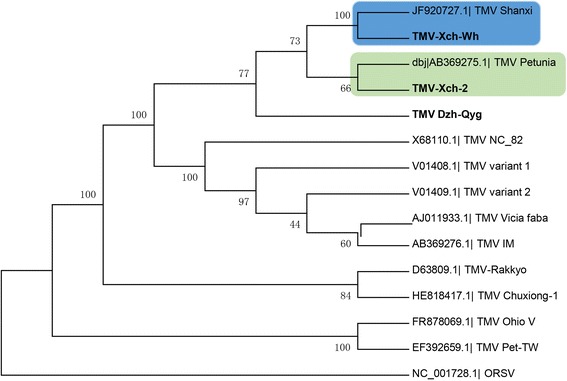
Phylogenetic analyses of Tobacco mosaic virus isolates and selected strains, with aligned nucleotide sequences, generated using the neighbor-joining method and MEGA6 software. The percentage of replicate trees in which the associated taxa clustered together in the bootstrap test (1000 replicates) is shown next to the branches. The tree was rooted using *Odontoglossum ring spot virus* (ORSV) as an out group sequence for the phylogenetic analysis

As for the Potyviruses, 5 isolates formed the same clade as the potato virus Y (NC_001616.1). The strains of Potato virus Y are divided into three groups (PVY^C^, PVY^O^, PVY^N^) [28, 29] and recombinant variants (PVY^NTN^) according to biological properties and genome sequence [30, 31]. PVY isolates Bzh-1 (KX650860), Bzh- 3 (KX650861), Bzh-4 (KX650862) and Xch-Wh (KX650859) clusters within the NTN group while the PVY-Xch-1 (KX650858) isolate falls within the PVY O strain group (Fig. 3). PVY-Xch-1 (KX650858) is closest to the PVY-Oz isolate (EF026074) from the USA with 90 % nucleotide identity. The PVY isolates have shown great ability to adapt to different changing environmental conditions throughout the world probably because of the genetically diverse populations of the RNA viruses [32]. Most PVY isolates are clustered in the PVY^NTN^ subgroup which has been regarded as the group of recombinant variant of PVY^N^. This can be explained by a 94 % nucleotide identity shared between PVY-Bzh-1 and PVY Guiding. The development of new variants through mutation and recombination had foster global distribution of the isolates [33]. The phylogenetic analysis of the TMV isolates also confirms the redistribution of the plant viruses across borders. TMV-Xch-2 (KX650854) form cluster with the Pertunia isolate (AB369275) from South Korea while TMV-Xch-Wh (KX650855) is clustered with Shanxi (JF920727) also from China. However, TMV-Dzh-Qyg (KX650856) is rooted independently in the tree (Fig. 4) forming a branch leading to the clade but closest to the Pertunia and Shanxi isolates as well, sharing 85 % nucleotide identity respectively.

The Chilli venial mottle virus isolate CVMV-Dzh-Qyg (KX650863) and PeMV-Xch (KX650857) identified in this study has 93 % and 94.18 % nucleotide identity with isolate Yp8 and PepMoVgp1 (NC_001517) respectively. The partial sequence of a Brassica yellow virus isolate BrYV-Xch-Wh clustered with the Brassica yellow virus (NC_016038.1) belonging to the genus Polerovirus. The analysis suggest that reassortment may have been a major event in the evolution of some of the strains and are derived from common recent ancestors that had passed through a bottleneck event. Phylogenetic analysis showed that, among the 22 isolates described worldwide, 14 Chinese isolates clustered together into an independent clade based on the near-complete genome nucleotide.

### Recombination events

The major source of variability in RNA viruses are mutation, re-assortment and recombination [34] which can result in insertion of unrelated sequence elements, as well as exchange, duplication or deletion of existing viral sequence elements. Segmental recombination events of the isolates were analyzed based on at least four positive statistical methods implemented in the RDP4 program used for the analysis. Recombination breakpoints detected by the methods were recognized in the cucumovirus and potyvirus strains (Additional file 1: Figure S7a and b). Some of the new isolates were predicted to have experienced recombination events supported by at least four programs. The major parent of PVY-Xch-Wh (KX650859) is SCRI-O isolate from United Kingdom while the minor parent is LYE84.2 from Spain. Recombination analysis in other isolates such as the PVY-Bzh-3 (KX650861) detected that the major and minor parent is WA-13 quan (China) and NE-11 (USA) isolate respectively while the PVY-Bzh-3 isolate was determined to be a recombinant of PVY-Bzh-4 and PVY-Xch-Wh. Fewer break points were observed in the cucumber virus isolates. The LS strain and FNY (USA) are the major and minor parent for CMV-Bzh-2 (KX650850) while other isolates share their origin from the Ly strain from Australia (Additional file 1: Figure S7a).

## Discussion

In spite of the different plant disease control mechanisms, plant virus still cause significant economic losses in tobacco production every year in China [14]. The effectiveness of disease control strategies can be affected by the genetic exchange and changes in composition of virus population [35]. Therefore, prompt identification of invading plant virus, elucidation of the molecular determinants and genetic diversities involved in pathogenesis is important to better understand plant–pathogen isolates. In this study, our goal was to survey tobacco plant in Anhui province of China for virus infection that caused devastating infection in the fields and also capture the genetic diversity and molecular variability of the different isolates identified across the province as well as determine the effectiveness of the application of next generation sequencing technology coupled with molecular techniques in discovery of plant viruses, without the prior knowledge of the virus.

We describe a bioinformatics pipeline to efficiently identify viruses in a mixed infection of tobacco and to differentiate different strains infecting the plant across the province. The bioinformatics method is based on the deep sequencing and *de novo* assembly of siRNAs. The assembled contigs generated from nine sRNA libraries were analyzed for the identification of the viruses associated with Tobacco. We determined the genome sequences of 22 isolates of plant viruses infecting tobacco collected from various regions across Anhui Province of China and validated through RT-PCR and Sanger sequencing. These identified viruses consist of 7 isolates of Cucumber Mosaic Virus, 5 isolates of Potato Virus Y, 3 isolates of Tobacco Mosaic Virus, 3 isolates of Tobacco Vein Banding Mosaic Virus, 1 isolate each of Pepper Mottle Virus, Brassica Yellow Virus, Chilli venial mottle virus, Broad Bean Wilt Virus 2 infecting Tobacco, a crop plant of paramount economic value. There are more isolates of CMV and PVY infecting Tobacco, compared to other identified isolates. This can be attributed to the diversity of the isolates [36] which has been reported in other parts of the world to be a serious concern to crop production.

Sequencing of the libraries of small RNA isolated from infected leaves shed more light on the consortium of replicating virus in the plant sample and proved decisive for the identification of the novel isolates [7, 9]. *De novo* assembly of the siRNA and BLAST search of assembled contigs to the non-redundant nucleotide and protein database identified virus sequence with more than 90 % similarity. To elucidate the molecular and genetic diversity of the isolates in Anhui province of China, we analyzed the isolates and sequences of previously reported recombinant and non-recombinant isolates [36–38] retrieved from the GenBank. Some of the isolates had experienced various recombination events which are similar to other strains from other parts of the world. They were clustered in the same subgroup with other strains of viruses prevalent in other parts of the world [36, 38, 39]. The CMV isolates identified in this study showed that subgroup I is of greater prevalence than subgroup II in China. The detected several subgroup IB isolates among historic CMV isolates and phylogenetic analysis further revealed presence of this specific subgroup in other parts of the world [31]. PVY is also considered as one of the most dangerous plant virus with different strains causing about 80 % of plant losses [23] which are dependent on infecting strains, time of infection and co-infecting species. The recombination events in plants plays a critical role in the virulence of plant viruses by generating genetic variation and producing new viruses [40]. The designation of PVY strain groups is based on the biological differences of the PVY strains to overcome resistance genes in tobacco and also allow the invasion of other plant viruses by suppressing the immune response of the plant at different strain groups.

Viral evolution and host adaptation are best understood by examining the role of recombination in generating and eliminating variation in viral sequences. RNA viral replicates, apparently lack proof-reading ability and as a consequence, the frequency of mutations is much higher than in organisms with a DNA genome [41]. The recombination events in some of the isolates are as a result of mutation and genetic reassortment which has been previously reported in other isolates [36, 42, 43].

The coverage, dispersal and complexity of virus population detected in this study, calls for a need for a constant survey of not only symptomatic crops but also other crops used in mixed cropping, and proper monitoring of disease spread and efficient management. Also a fast and efficient detection method, as the Next generation sequencing that do not need a prior knowledge of the virus should be employed to identify viruses. Deep sequencing, bioinformatics and phylogenetic analysis, as well as comparison of the different virus species identified in Tobacco presents an important revelation of molecular variability of viruses causing devastating effects on the crop. Furthermore the proliferation of new genetic types signals a high risk for crops that must be addressed with efficient viral control and diagnostic methods.

## Conclusion

In this study we describe the discovery of a consortium of plant viruses infecting Tobacco that are broadly distributed in Anhui province of China. We further characterized the genome of the 22 isolates, its variability and the siRNAs induced in tobacco plant in response to virus infection. Our result showed the effectiveness of the custom made bioinformatics pipeline coupled with molecular techniques and phylogenetic analysis, in diagnostics and identification of plant virus. Survey of plant viruses and prompt diagnostics should be frequently carried out in areas known for large cultivation of economically important crops.

## Methods

### Collection and preparation of samples

**Fig. 5:**
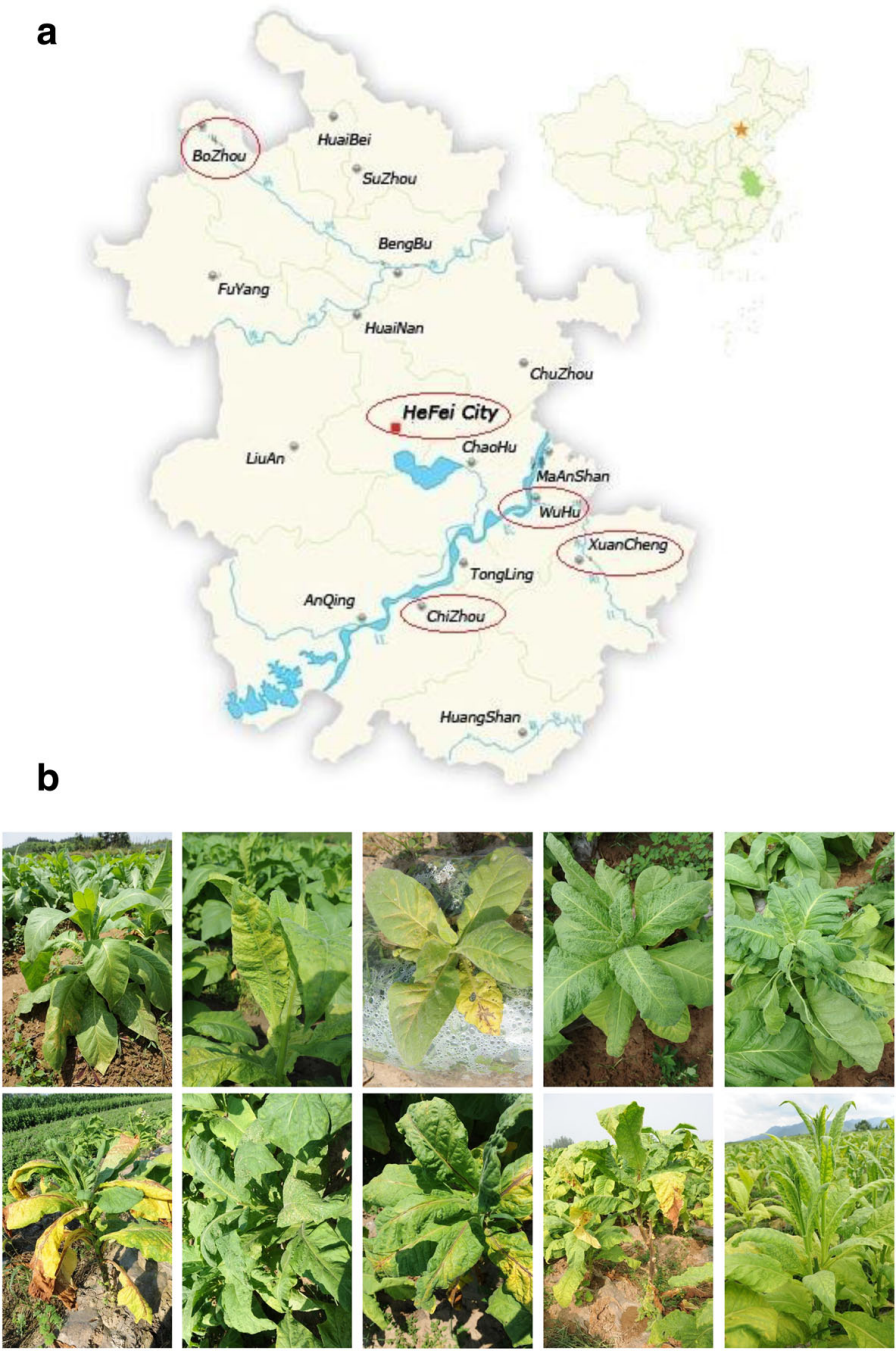
**a** Sampling areas of infected tobacco plants in Anhui Province of China. **b** Typical view representation of yellow mosaic mosaic, stunting, Shoestring and deformation symptom of the overall Tobacco leaf samples collected across Anhui Province

A field survey of the potential viral pathogens of Tobacco was conducted across farm fields in Anhui Province of China (Fig. 5a). One hundred and four symptomatic (mosaic, mottling, yellowing, ring spots, stunting, Shoestring and deformation)(Fig. 5b) leaf samples of cultivated Tobacco (*Nicotina tobaccum*) were collected from different regions (Xuancheng, Wuhu, Dongzhi, Qingyang, Bozhou) of Anhui province. (Tables 1). Leaf samples were immediately frozen in liquid nitrogen and stored at −80 °C until RNA extraction.

### Preparation of total RNA, small RNA library construction and sequencing

Total RNA was extracted from leaf samples as earlier described [16]. Briefly, the frozen leaves of symptomatic samples were pulverized in liquid nitrogen and suspended in 1.5 ml tube containing extraction buffer (50 mM Tris–HCl, pH 8.0; 150 mM LiCl; 5 mM EDTA, pH 8.0; 5 % SDS, 5 % PVP). The supernatant was treated with 2.5 M Potassium Acetate (pH 4.8) followed by Chloroform : isoamyl alcohol (24:1 v/v). The pellets were washed in 70 % ethanol, air dried and re-suspended in nuclease-free water. Integrity of the RNA was verified in ethidium-bromide stained 1.2 % agarose gels after electrophoresis, and purity was assessed by measuring the absorbance ratio (1.8–2.0) at A_260_/A_280_ nm using a Eppendorf BioPhotometer Plus (Germany). Total RNA from symptomatic leaf samples were pooled for small RNA library construction based on the site of collection as previously described [44] and deep sequencing was performed on an Illumina HiSeq-2000 sequencing platform (BGI-ShenZhen, China) using the manufacturer’s instruction for sequencing.

### Bioinformatics analysis

The sRNA sequence processing, assembly, and virus genome identification were conducted using a custom bioinformatics pipeline (Fig. 2). Briefly, quality of the raw Illumina sRNA reads were screened using the fastqc (http://www.bioinformatics.babraham.ac.uk/projects/fastqc) and the adapter sequence trimmed with the Cutadapt software (version 1.3) (https://cutadapt.readthedocs.io/en/stable/). Low quality reads were cleaned with an in-house Perl script. Trimmed sRNA sequences shorter than 18 nt and longer than 30 nt were discarded. The clean sRNAs reads from each sample were assembled *de novo* into contigs based on *De Bruijin* graphs with a *k-mer* length of 17 using Velvet [18] and Oases program downloaded from the European Bioinformatics Institute (http://www.ebi.ac.uk/~zerbino/oases/). The assembled contigs were used to query the non-redundant GenBank nucleotide and protein databases, respectively, using the BLAST program [45]. Highest hits having ≥ 90 % coverage and identity with the nucleotide sequences in the non-redundant NCBI database were extracted for further analysis using BLASTn (e-value cutoff: 10^−3^). Furthermore, they were filtered and searched against the non-redundant protein database of NCBI by BLASTx and tblastx to compare the homology, as short sequences may lead to false positive results [46]. Final hits with nucleic acid and protein coverage ≥ 90 % and identity ≥ 90 % transcription of the virus were identified and aligned to references in order to determine their orientation and positions. The small RNAs of the hits were mapped to the to the closest distantly related viral genome with Bowtie [20] to determine the distribution and abundance of the sRNA.

### RT-PCR, cloning, full-length genome amplification, and sequencing

Reverse transcription PCR (RT-PCR) was performed using primers (Table S1) designed from sequences of specific assembled contigs to bridge the gaps between them, so as to obtain the near-complete genome sequences. The PCR product segments were electrophoresed in 1.0 % agarose gels, bands were excised and purified using the SanPrep Gel Extraction Kit (Sangon Biotech, China). The amplification products were ligated to pMD18-T vector (Takara Biotechnology Co., Ltd), transformed into *Escherichia coli* DH5α and positive clones were sequenced. Two clones from independent PCR reactions products were sequenced twice on both strands for each amplified fragment through the conventional Sanger dideoxy sequencing.

### Phylogenetic analysis

ORFs were predicted using ORF Finder. Conserved domains/motifs were analyzed using SMART [12]. The new sequences were aligned with other sequences retrieved from the GenBank with Mafft [47] and phylogenetic analyses were conducted using the neighbor-joining method by MEGA6 [48], with the bootstrap of 1000 replicates [49]. Maximum likelihood resulted in similar phylogenetic trees. Other isolates were retrieved from the GenBank database for the phylogenetic analysis. Origin and accession number of the isolates are listed in the Additional file 2: Table S2, S3 and S4.

### Recombination analysis

The putative recombination events among the isolates and genus member were evaluated using the Recombination Detection Program v.4.66 (RDP4) [50] made up of different methods namely recombination detection program (RDP), statistical tests for detecting gene conversion (GENECONV) [51], BOOTSCAN [52], MaxiChi [53], Chimera [54], SiScan [55], and 3SEQ [56], implemented in the RDP Beta 4.66 program. Alignments of nucleotide sequences produced in Mafft [47] and MEGA6 [48] were used in RDP4 with the default settings (0.05 *p*-value cut-off). Recombination events were considered as significant if more than four algorithms had a consensus *P*-value ≤ 0.01, in addition to phylogenetic evidence of recombination. Otherwise, they were regarded as ‘tentative’ recombination events [57].

## Additional files

Additional file 1: Figure S1. 21 and 24 nt peak distribution of the libraries. **Figure S2 (a,b,c).** Length distribution of total sRNAs (18 to 30 nt) in the nine libraries. **Figure S3.** Distribution of siRNA Reads on Reference Genome shows coverage across the reference. **Figure S4.** Phylogenetic analyses of isolates of the genus Potyvirus and some selected group members based on complete genome sequences, generated using the neighbor-joining method and MEGA6 software. The percentage of replicate trees in which the associated taxa clustered together in the bootstrap test (1000 replicates) is shown next to the branches. **Figure S5.** Phylogenetic analyses of CMV Isolates and selected cucumoviruses based on complete genome sequences, generated using the neighbor-joining method and MEGA6 software. The percentage of replicate trees in which the associated taxa clustered together in the bootstrap test (1000 replicates) is shown next to the branches. **Figure S6.** Phylogenetic analyses of isolates of the genus Poleroviruss (A) and Tobamovirus (B) based on complete genome sequences, generated using the neighbor-joining method and MEGA6 software. The percentage of replicate trees in which the associated taxa clustered together in the bootstrap test (1000 replicates) is shown next to the branches. **Figure S7a and b.** Putative recombination event involving (a) Potato virus Y and (b) Cucumber mosaic virus isolates from Tobacco calculated by Recombination Detection Program v. 4.16. (PPTX 466 kb)
Additional file 2: Table S1. Primer sets used in this study for RT-PCR to bridge the gaps between the assembled contigs of each isolates. **Table S2.** Strain, accession numbers and origin of reported Cucumber mosaic virus isolates used for phylogenetic comparison of sequence in this study. **Table S3.** Strain, accession numbers and origin of reported Potato virus Y isolates used for phylogenetic comparison of sequence in this study. **Table S4.** Strain, accession numbers and origin of reported TMV isolates retrieved from Genbank used for phylogenetic comparison and recombination analysis of sequence in this study. (DOCX 25 kb)

## Abbreviations

Ago: Argonaut protein
BBWV: *Broad bean wilt virus 2*
BrYV: *Brassica yellow virus*
CMV: *Cucumber mosaic virus*
CVMV: *Chilli venial mottle virus*
DCL: Dicer-like ribonuclease
dsRNA: double stranded RNA
ELISA: Enzyme-linked immunosorbent assay
NGS: Next Generation Sequencing
PCR: Polymerase chain reaction
PeMV: *Pepper mottle virus*
PVY: *Potato virus Y*
RISC: RNA-induced silencing complex
siRNAs: small-interfering RNAs
TMV: *Tobacco mosaic virus*
TVBMV: *Tobacco vein banding Mosaic virus*
vsRNAs: viral small RNAs
