# Supplementary material for: Ecogenomic survey of plant viruses infecting Tobacco by Next generation sequencing
Source: Virol J. 2016 Nov 4;13:181. doi: 10.1186/s12985-016-0639-7 (PMC5096307; doi:10.1186/s12985-016-0639-7)
Supplement: Additional file 1: Figure S1. — 21 and 24 nt peak distribution of the libraries. Figure S2 (a,b,c). Length distribution of total sRNAs (18 to 30 nt) in the nine libraries. Figure S3. Distribution of siRNA Reads on Reference Genome shows coverage across the reference. Figure S4. Phylogenetic analyses of isolates of the genus Potyvirus and some selected group members based on complete genome sequences, generated using the neighbor-joining method and MEGA6 software. The percentage of replicate trees in which the associated taxa clustered together in the bootstrap test (1000 replicates) is shown next to the branches. Figure S5. Phylogenetic analyses of CMV Isolates and selected cucumoviruses based on complete genome sequences, generated using the neighbor-joining method and MEGA6 software. The percentage of replicate trees in which the associated taxa clustered together in the bootstrap test (1000 replicates) is shown next to the branches. Figure S6. Phylogenetic analyses of isolates of the genus Poleroviruss (A) and Tobamovirus (B) based on complete genome sequences, generated using the neighbor-joining method and MEGA6 software. The percentage of replicate trees in which the associated taxa clustered together in the bootstrap test (1000 replicates) is shown next to the branches. Figure S7a and b. Putative recombination event involving (a) Potato virus Y and (b) Cucumber mosaic virus isolates from Tobacco calculated by Recombination Detection Program v. 4.16. (PPTX 466 kb) [file 12985_2016_639_MOESM1_ESM.pptx]

## Slide 1
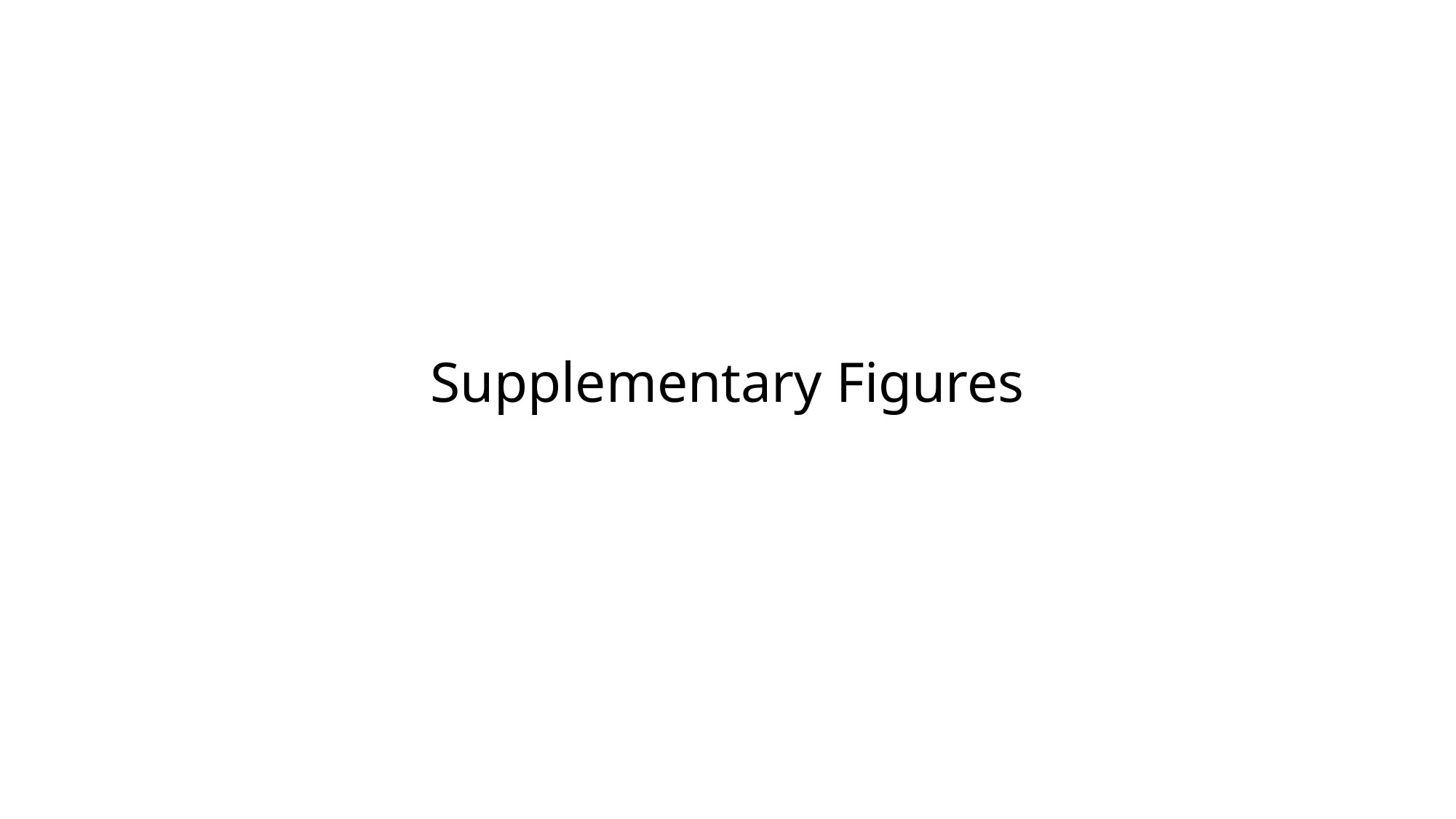

# Supplementary Figures

## Slide 2
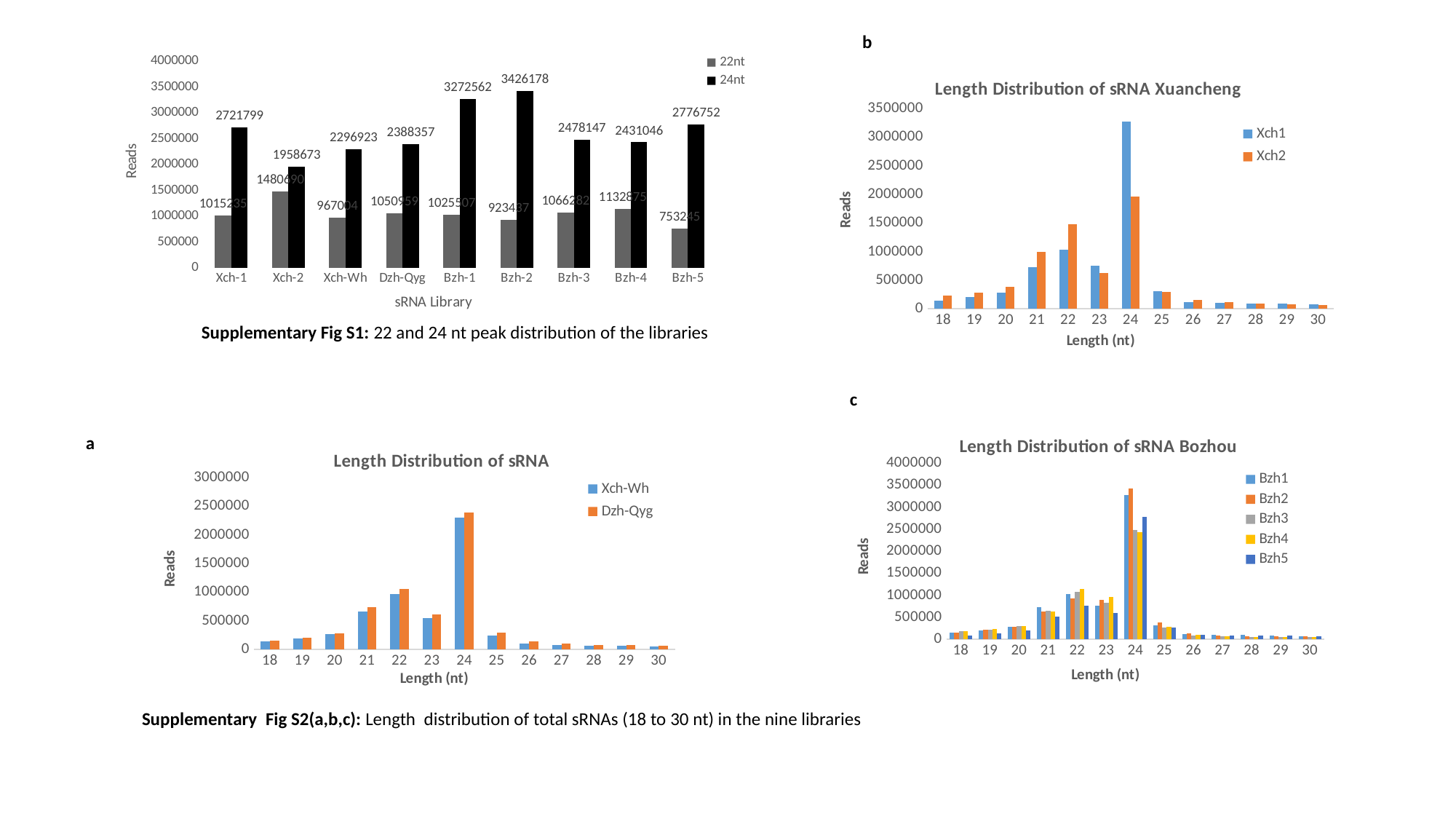

b
### Chart
| Category | | |
|---|---|---|
| Xch-1 | 1015235.0 | 2721799.0 |
| Xch-2 | 1480690.0 | 1958673.0 |
| Xch-Wh | 967004.0 | 2296923.0 |
| Dzh-Qyg | 1050959.0 | 2388357.0 |
| Bzh-1 | 1025507.0 | 3272562.0 |
| Bzh-2 | 923437.0 | 3426178.0 |
| Bzh-3 | 1066282.0 | 2478147.0 |
| Bzh-4 | 1132875.0 | 2431046.0 |
| Bzh-5 | 753245.0 | 2776752.0 |
### Chart: Length Distribution of sRNA Xuancheng
| Category | | |
|---|---|---|
| 18 | 147171.0 | 231899.0 |
| 19 | 200660.0 | 283975.0 |
| 20 | 284625.0 | 383920.0 |
| 21 | 728389.0 | 989143.0 |
| 22 | 1025507.0 | 1480690.0 |
| 23 | 757244.0 | 630850.0 |
| 24 | 3272562.0 | 1958673.0 |
| 25 | 311399.0 | 295767.0 |
| 26 | 119874.0 | 153321.0 |
| 27 | 97740.0 | 110981.0 |
| 28 | 92032.0 | 87804.0 |
| 29 | 84383.0 | 74309.0 |
| 30 | 71515.0 | 64506.0 |Supplementary Fig S1: 22 and 24 nt peak distribution of the libraries
c
### Chart: Length Distribution of sRNA Bozhou
| Category | | | | | |
|---|---|---|---|---|---|
| 18 | 147171.0 | 150005.0 | 173836.0 | 180652.0 | 87529.0 |
| 19 | 200660.0 | 212846.0 | 221638.0 | 235873.0 | 126426.0 |
| 20 | 284625.0 | 285197.0 | 300881.0 | 302303.0 | 191469.0 |
| 21 | 728389.0 | 621358.0 | 648189.0 | 629838.0 | 510750.0 |
| 22 | 1025507.0 | 923437.0 | 1066282.0 | 1132875.0 | 753245.0 |
| 23 | 757244.0 | 897174.0 | 833553.0 | 952235.0 | 587917.0 |
| 24 | 3272562.0 | 3426178.0 | 2478147.0 | 2431046.0 | 2776752.0 |
| 25 | 311399.0 | 387735.0 | 257793.0 | 284669.0 | 270627.0 |
| 26 | 119874.0 | 128914.0 | 81614.0 | 93659.0 | 98652.0 |
| 27 | 97740.0 | 86814.0 | 62081.0 | 66324.0 | 84129.0 |
| 28 | 92032.0 | 71869.0 | 54520.0 | 56365.0 | 78517.0 |
| 29 | 84383.0 | 64230.0 | 49796.0 | 50907.0 | 75964.0 |
| 30 | 71515.0 | 57940.0 | 44536.0 | 45246.0 | 71034.0 |a
### Chart: Length Distribution of sRNA
| Category | | |
|---|---|---|
| 18 | 144419.0 | 158289.0 |
| 19 | 194742.0 | 198945.0 |
| 20 | 271815.0 | 275816.0 |
| 21 | 656980.0 | 736010.0 |
| 22 | 967004.0 | 1050959.0 |
| 23 | 550662.0 | 604813.0 |
| 24 | 2296923.0 | 2388357.0 |
| 25 | 235990.0 | 297239.0 |
| 26 | 100968.0 | 138109.0 |
| 27 | 80955.0 | 100883.0 |
| 28 | 70397.0 | 82105.0 |
| 29 | 62708.0 | 71874.0 |
| 30 | 55306.0 | 63835.0 |Supplementary Fig S2(a,b,c): Length distribution of total sRNAs (18 to 30 nt) in the nine libraries

## Slide 3
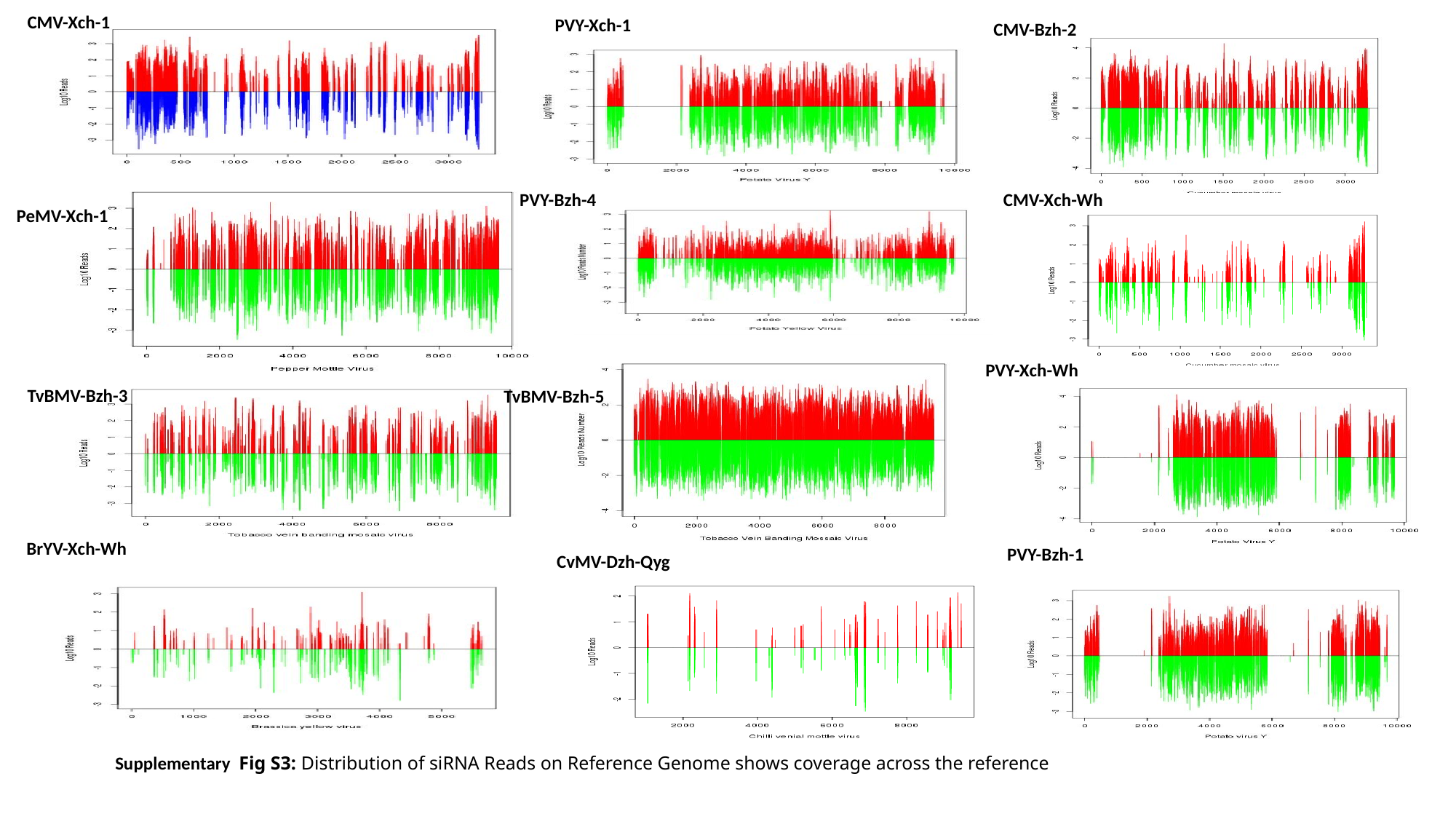

CMV-Xch-1
PVY-Xch-1
CMV-Bzh-2
PVY-Bzh-4
CMV-Xch-Wh
PeMV-Xch-1
PVY-Xch-Wh
TvBMV-Bzh-3
TvBMV-Bzh-5
BrYV-Xch-Wh
PVY-Bzh-1
CvMV-Dzh-Qyg
# Supplementary Fig S3: Distribution of siRNA Reads on Reference Genome shows coverage across the reference

## Slide 4
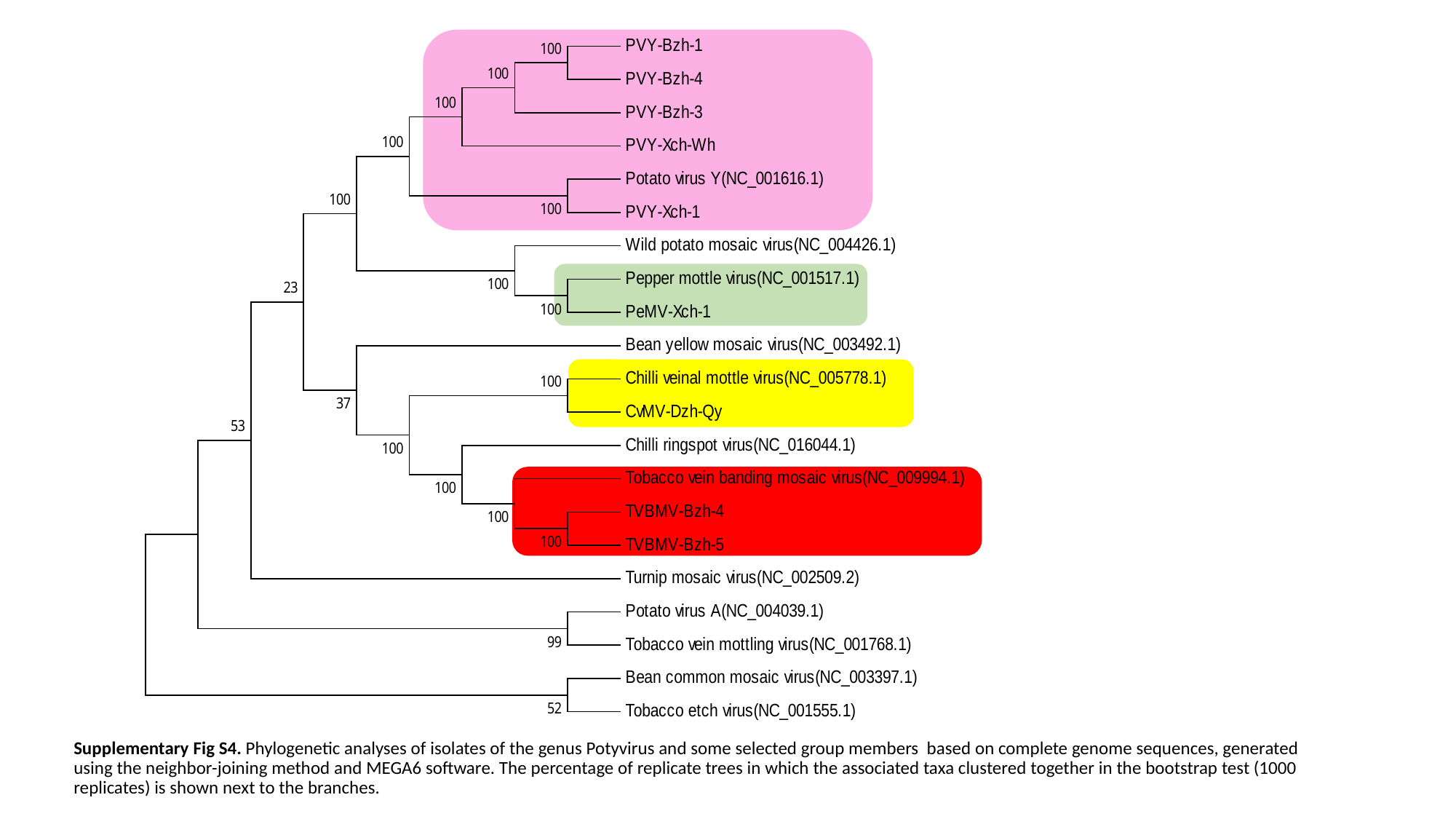

Supplementary Fig S4. Phylogenetic analyses of isolates of the genus Potyvirus and some selected group members based on complete genome sequences, generated using the neighbor-joining method and MEGA6 software. The percentage of replicate trees in which the associated taxa clustered together in the bootstrap test (1000 replicates) is shown next to the branches.

## Slide 5
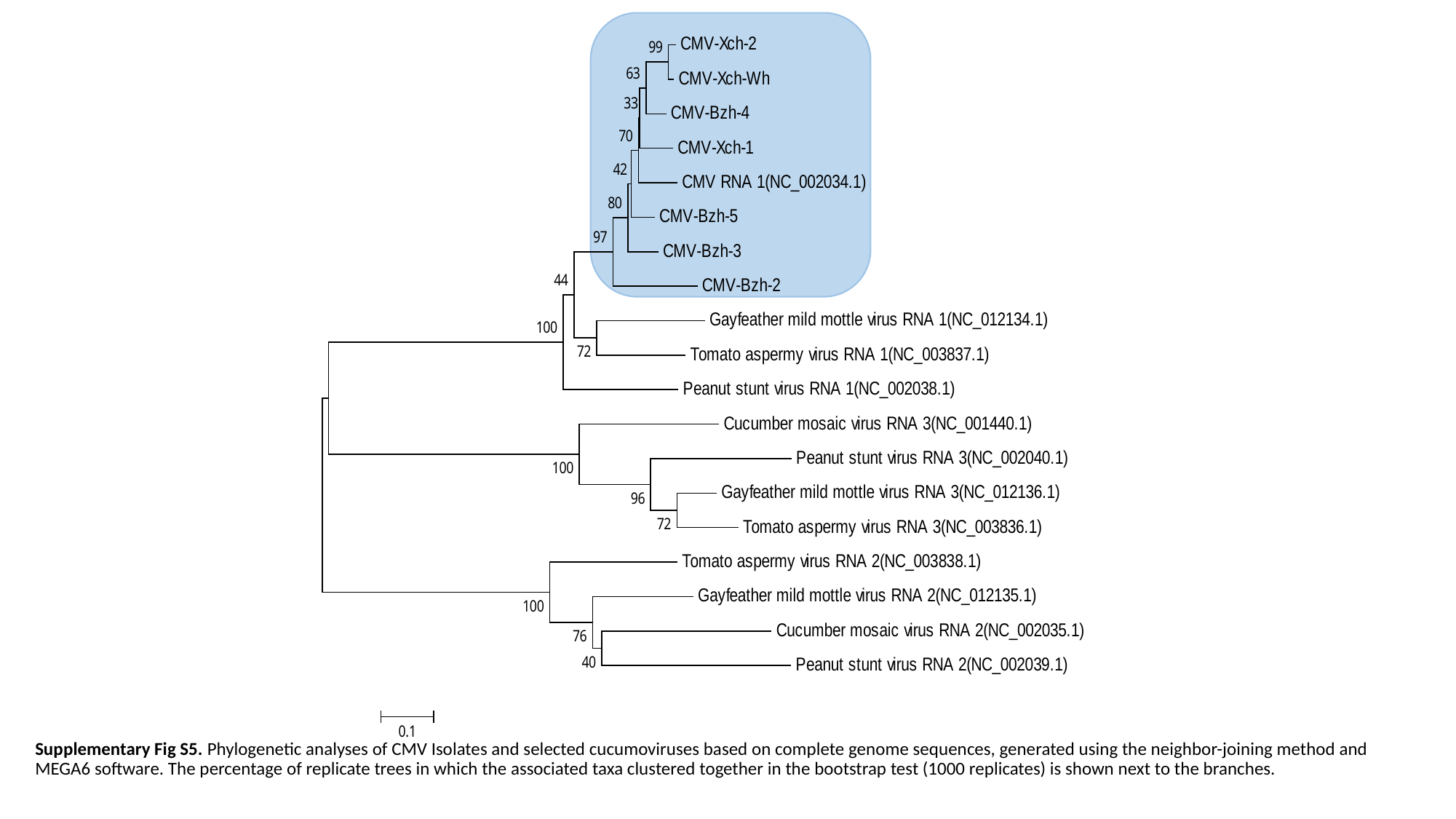

Supplementary Fig S5. Phylogenetic analyses of CMV Isolates and selected cucumoviruses based on complete genome sequences, generated using the neighbor-joining method and MEGA6 software. The percentage of replicate trees in which the associated taxa clustered together in the bootstrap test (1000 replicates) is shown next to the branches.

## Slide 6
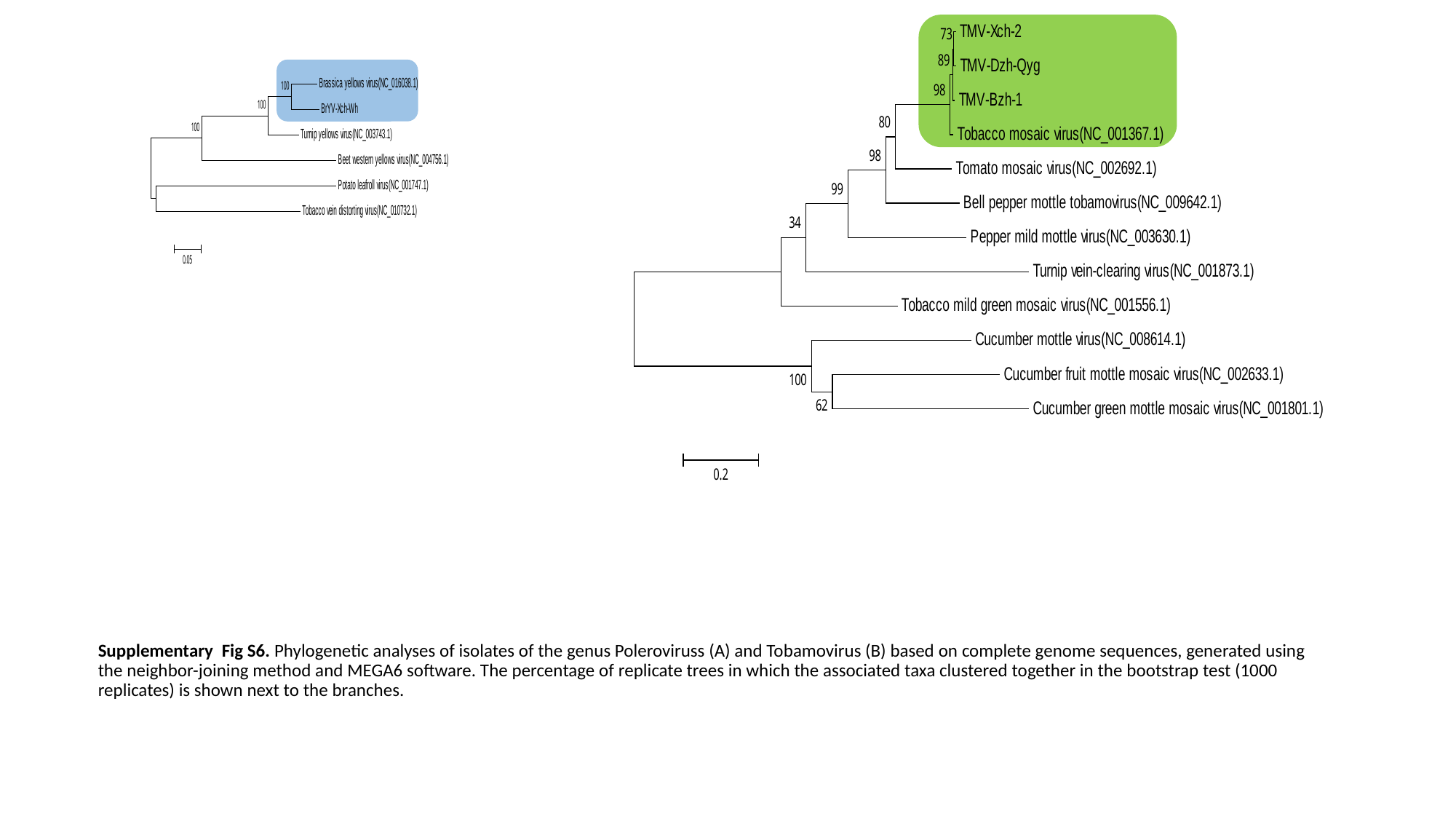

Supplementary Fig S6. Phylogenetic analyses of isolates of the genus Poleroviruss (A) and Tobamovirus (B) based on complete genome sequences, generated using the neighbor-joining method and MEGA6 software. The percentage of replicate trees in which the associated taxa clustered together in the bootstrap test (1000 replicates) is shown next to the branches.

## Slide 7
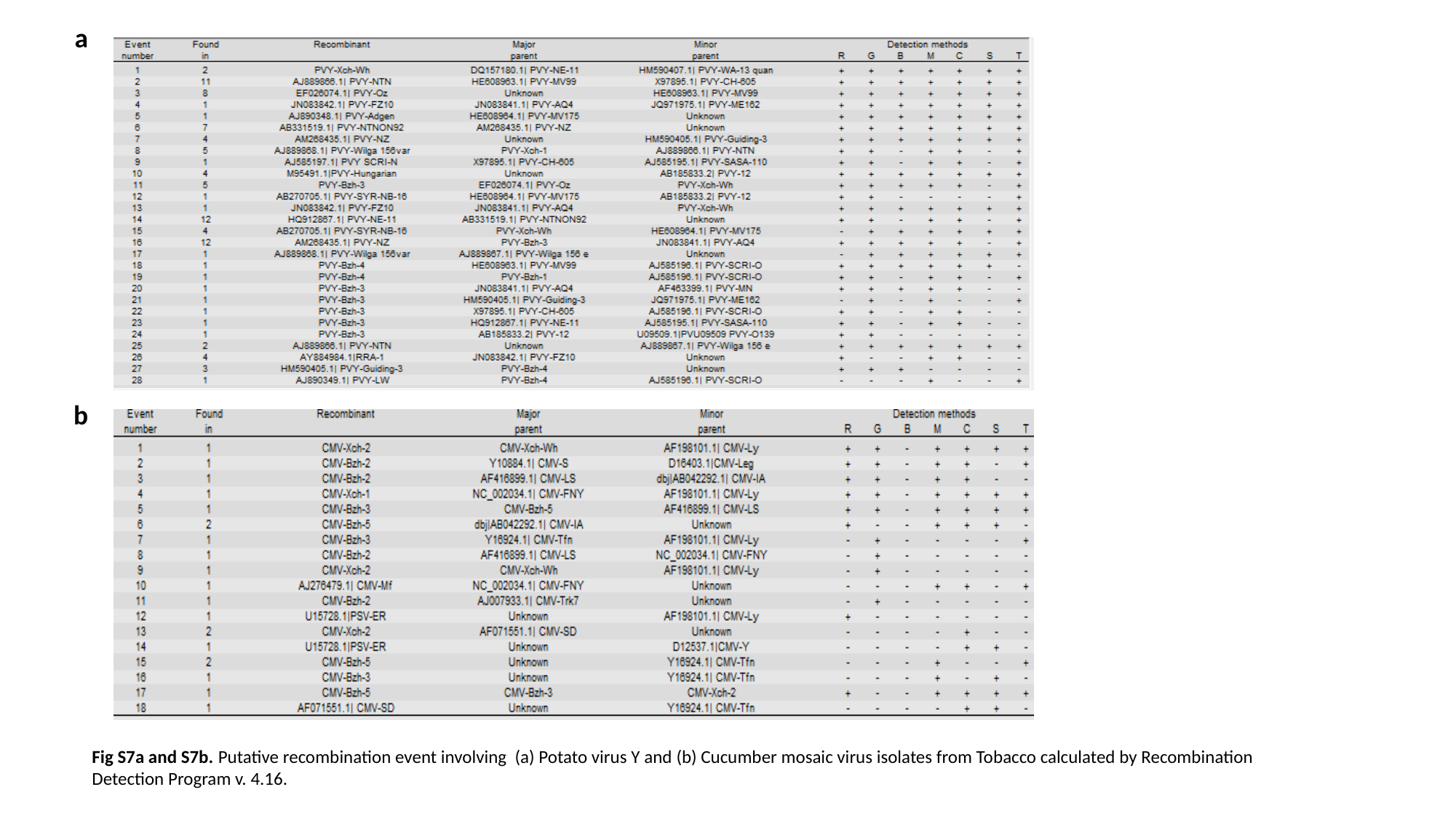

a
b
Fig S7a and S7b. Putative recombination event involving (a) Potato virus Y and (b) Cucumber mosaic virus isolates from Tobacco calculated by Recombination Detection Program v. 4.16.
