# Supplementary material for: Ecogenomic survey of plant viruses infecting Tobacco by Next generation sequencing
Source: Virol J. 2016 Nov 4;13:181. doi: 10.1186/s12985-016-0639-7 (PMC5096307; doi:10.1186/s12985-016-0639-7)
Supplement: Additional file 2: Table S1. — Primer sets used in this study for RT-PCR to bridge the gaps between the assembled contigs of each isolates. Table S2. Strain, accession numbers and origin of reported Cucumber mosaic virus isolates used for phylogenetic comparison of sequence in this study. Table S3. Strain, accession numbers and origin of reported Potato virus Y isolates used for phylogenetic comparison of sequence in this study. Table S4. Strain, accession numbers and origin of reported TMV isolates retrieved from Genbank used for phylogenetic comparison and recombination analysis of sequence in this study. (DOCX 25 kb) [file 12985_2016_639_MOESM2_ESM.docx]

**Table S1:** Primer sets used in this study for RT-PCR to bridge the gaps between the assembled contigs of each isolates

| **Library** | **Used for Clone** | **Primer Sequence (5’ 3’)** |  |
| --- | --- | --- | --- |
| **Xch 1** | **CMV-Xch-1** | 15493F GGAGGAAGATTAGGAGTGCT | 22404R ACGCAACGCTCAATACCA |
|  |  | 22404F CCGAGGGATGACTTATGG | 6267R TTCCACTCAACCAAGTCCTG |
|  |  | 830F CAAGAAATCCGCCCAAGA | 4427R CCAAAACATATAGCCCGCAC |
|  |  | 4427F GCGACGCCTCATTTGATA | 18492R TCGAAGTACACGAAATGGGT |
|  | **PVY-Xch-1** | 7923F CAAGCCTTGAAGTTTCGC | 20091R TCTATTTGCGCCCCAGTG |
|  | **PeMV-Xch** | 5627F GCAATAACAGTGGGCAACC | 4154R TTCCCGCATTCACATCAG |
| **Xch 2** | **CMV-Xch-2** | 11282F GACTTCTGCACTCATTCGG | 2998R ACAACGGCGTGACATCTT |
|  |  | 2998F TCGTACCGAGGGATGACA | 891R GTTCCATCCACGCTTTCT |
|  |  | 7940F GCGACCGCTATGCTTGAA | 11240R TCACAAGGGCGTCAACAG |
|  | **TMV-Xch-2** | 951F AGGTCCGAATGGGATGTG | 1525R CACCGATAACTCCGAAAG |
|  |  | 1431F GTGGCGATGTCATTGTGC | 558R TGATGGAGACTGGTGTAGGG |
|  |  | 666F CGCCAAAGACTGGTGATA | 288R CGAGCTGGCCTATTGTTA |
|  |  | 3342F TGGTGACGATAGTCTGCTGT | 402R AACGAAACAGCAACATCACA |
|  |  | 402F TGTGATGTTGCTGTTTCGTT | 1224R AACGAAACAGCAACATCACA |
| **Xch-Wh** | **CMV-Xch-Wh** | 2340F CCAGTACCACTTCGCTATGC | 2375R TGGTCCACTGATTCCTCTTG |
|  |  | 6456F AGAGAGCCCAAGGTTAGTGA | 2495R CGTATCAATCCAGTGCCTC |
|  | **BrYV-Xch-Wh** | 6068F TCAATCTGGACTAGAAGCGC | 861nR TCTGTCTCGCCTTGCTGT |
|  |  | 1342nR AGAAAGATGGCTGGGTCA | 7600R GGGATTGGTGCCATTAGA |
|  |  | 4639nF GAGCACTGACCTGGAACAA | 1029nR GCTTTGCTTGCTTGTGGG |
|  |  | 1029nF CCGCTACCGCCTCATTAT | 1017nR TGTAAACGACCTCCTCCC |
|  |  | 3931nF GATGCCCGCTGGTATTCA | 4385nR ATTTCCATATCAGGGTGCCC |
|  | **TMV-Xch-Wh** | 445F GCATCTGTTCAAGGGACG | 1339R CGAACTCATCAGCGGGTA |
|  |  | 747F CGGAGACACACAGCAGATTC | 275R CAGAGTATGTCTCGCCTTGC |
|  |  | 78F CCGAGTTGTCTGGCATCA | 1128R ACTGCACAGTGGAATTCATT |
|  |  | 344F TGGAAGATGTCCCTATGTCA | 30R AACTGATTTCCTAAGGCATT |
| **Dzh-Qyg** | **TMV-Dzh-Qyg** | 916F GGAGTTTGTGTCGGTGTGTA | 779R GTTACTTGCGGTGAAGGTTT |
| **Bzh-3** | **CMV-Bzh-3** | 9957F CTGACGGACCAGACGTACTC | 21625R TCGCCAGACTGCATAGACAT |
|  |  | 21625F GCAGTCTGGCGAACGAAT | 21986R TTTCCCATCGGTAACAGC |
|  |  | 9492F TTGGCCGAGGAGAAAGCT | 21990R TCATCACCACCCGCCATA |
|  |  | 13633F GCTCCAACCGGAACTATCAG | 6385R TGGGCTACGGAATGTGGT |
|  | **PVY-Bzh-3** | 3167F CGCCAAAGTTGACAGAGG | 3412R TTCCACTTGTCCGTCCTG |
|  |  | 7268F CAGGGGTGAGTAAGAAGTGC | 8045R TTTGATCACCGCTGTTACCT |
|  |  | 19193F CCTATTCAAGGCGGGTTT | 4409R GCCCTTCCTGTGGGTTTT |
|  |  | 4409F TGAGATTGTCTGCATGGAGC | 34269R ATGGTCCAGTGAGGAAAGG |
| **Bzh-4** | **PVY-Bzh-4** | 14944F TCCAGATGGCAGCTCCTAGT | 10494R ATTGAGCCACCTGCCATT |
|  |  | 3398F CCACAGGAAGGGCAGATT | 7965R CGTTGGTGTCGCACATCATA |
|  |  | 14327F GCTTTGGGAAACGGTTGT | 7082R TCTTCCAAGTCCCCTTCAAC |
| **Bzh-5** | **CMV-Bzh-5** | 18222R CGGCGAGCGTATAGATATCA | 12010R AGTAAACGATTCCCTCAGGC |
|  |  | 10647F GTCACCCGTTCACTACAAGC | 9222R ATTGACCAAAGTGCAGCAGC |
|  | **BBWV2-Bzh-5** | 5452F AAGGCTTGATGGAAGAGGAC | 8517R GCTTGAAGACAAATTCCACG |

**Table S2:** Strain, accession numbers and origin of reported Cucumber mosaic virus isolates used for phylogenetic comparison of sequence in this study

| **Strain** | **Accession No** | **Origin** |
| --- | --- | --- |
| FNY | D00356 | United States (N.Y.) |
| IA | AB042292 | Indonesia |
| Ix | U20220 | Philippines |
| Leg | D16403 | Japan |
| LS | AF416899 | United States (N.Y.) |
| Ly | AF198101 | Australia |
| Mf | AJ276479 | South Korea |
| NT9 | D28778 | Taiwan |
| O | —*b* | Japan |
| Q | X02733 | Australia |
| S | Y10884 | South Africa |
| SD | AF071551 | China |
| Tfn | Y16924 | Italy |
| Trk7 | AJ007933 | Hungary |
| Y | D12537 | Japan |
| ER-PSV | U15728 | United States (Ky.) |
| Xch-1 | KX650847 | China |
| Xch-2 | KX650848 | China |
| Xch-Wh | KX650849 | China |
| Bzh-2 | KX650850 | China |
| Bzh-3 | KX650851 | China |
| Bzh-4 | KX650852 | China |
| Bzh-5 | KX650853 | China |

**Peanut stunt virus* ER (U15728.1) was used as an out group for the tree construction

**Table 3:** Strain, accession numbers and origin of reported Potato virus Y isolates used for phylogenetic comparison of sequence in this study

| **Strain** | **Accession No** | **Name** | **Origin** |
| --- | --- | --- | --- |
| NTN | AB185833 | 12 | Syria |
| N | AB270705 | SYR-NB-16 | Syria |
| NTN | AB331515 | NTND6 | Japan |
| NTN | AB331519 | NTNON92 | Japan |
| C | AF463399 | MN | Italy |
| O | AJ585195 | SASA-110 | UK |
| O | AJ585196 | SCRI-O | UK |
| N | AJ585197 | SCRI-N | UK |
| N | AJ585198 | SASA-61 | UK |
| NTN | AJ585342 | NIB-NTN | Slovenia |
| NTN | AJ889866 | 12-94 | Poland |
| N:O | AJ889868 | 156 var | Germany |
| N:O | AJ889867 | 156 | Germany |
| C | AJ890348 | Adgen-C | France |
| NTN | JN083841 | AQ4 | China |
| NTN | JN083842 | FZ10 | China |
| O | EF026074 | Oz | USA |
| O | U09509 | O-139 | Canada |
| N | X97895 | N605 | Switzerland |
| O | AJ890349 | LW | Germany |
| N | HM590405 | Guiding-3 | China |
| N | AY884983 | Mont | USA |
| N | DQ157180 | NE-11 | USA |
| NTN | HM590406 | HC-2 quan | China |
| O | HM590407 | WA-13 quan | China |
| N | HQ912867 | NE-11a | USA |
| NTN | M95491 | Hungarian | Hungarian |
| Unknown | HE608963 | MV99 | Germany |
| Unknown | HE608964 | MV175 | Germany |
| N | JQ971975 | ME-162 | China |
| N | AM268435 | Strain N (NZ) | New Zealand |
| C | EU563512 | PRI-509 | Netherlands |
| C | AJ439544 | SON41 | France |
| C | AJ439545 | LYE84.2 | Spain |
| N | AY884984 | RPA-1 | USA |
| O | KX650858 | PVY-Xch-1 | China |
| NTN | KX650859 | PVY-Xch-Wh | China |
| NTN | KX650860 | PVY-Bzh-1 | China |
| NTN | KX650861 | PVY-Bzh-3 | China |
| NTN | KX650862 | PVY-Bzh-4 | China |

* *Sunflower chlorotic mottle virus* (NC_014038.1) was used as an out group sequence for the phylogenetic analysis

**Table S4:** Strain, accession numbers and origin of reported TMV isolates retrieved from Genbank used for phylogenetic comparison and recombination analysis of sequence in this study

| **Strain** | **Accession No** | **Origin** |
| --- | --- | --- |
| Rakkyo | D63809 | Japan |
| Shanxi | JF920727 | China |
| Petunia | AB369275 | South Korea |
| NC_82 | X68110 | South Korea |
| Vicia faba | AJ011933 | China |
| Variant 1 | V01408 | Germany |
| Impatiens | AB369276 | Korea |
| Variant 2 | V01409 | USA |
| Pet-TW | EF392659 | Taiwan |
| Chuxiong-1 | HE818417 | China |
| TMV Ohio V | FR878069 | USA |
| Xch-2 | KX650854 | China |
| Xch-Wh | KX650855 | China |
| Dzh-Qyg | KX650857 | China |

^*^*Odontoglossum ringspot virus* (NC_001728.1), a member of the genus Tobamovirus, which was used as an out-group species
